# ERBB4 and Neurodegeneration: Association Between Hypothalamic–Pituitary–Adrenal (HPA) Axis: Associated Neurodegenerative Pathogenesis

**DOI:** 10.3390/cells15181710

**Published:** 2026-09-20

**Authors:** Eva Bagyinszky, Seong Soo A. An

**Affiliations:** 1 College of BioNano Technology, Gachon University, Seongnam 13120, Republic of Korea; eva85@gachon.ac.kr; 2 Department of Bionano Technology, Gachon Medical Research Institute, Gachon University, Seongnam 13120, Republic of Korea

**Keywords:** ERBB4, neureulin-1, HPA axis, mutation, neurodegenerative diseases

## Abstract

The hypothalamic–pituitary–adrenal (HPA) axis is the central neuroendocrine system that controls physiological stress responses and maintains homeostasis through the coordinated interactions among the hypothalamus, pituitary gland, and adrenal cortex. Dysregulation of the HPA axis is associated with stress-related psychiatric conditions and neurodegenerative diseases, including Alzheimer’s disease, Parkinson’s disease, and amyotrophic lateral sclerosis. Chronic stress creates a detrimental environment in the brain, inducing structural changes and accelerating brain aging and the loss of neurons. Receptor tyrosine-protein kinase ERBB4 (ERBB4), a member of the epidermal growth factor receptor (EGFR) family, is activated primarily by neuregulin ligands and plays an essential role in neuronal development, synaptic plasticity, and cell survival. Emerging evidence suggests that ERBB4 signaling influences neuroendocrine regulation and stress responsivity. While further studies are needed to provide direct mechanistic evidence linking ERBB4-mediated HPA axis dysregulation to neurodegenerative cell death, this manuscript critically evaluated the preclinical models and proposes a possible feed-forward framework wherein ERBB4 served as a permissive homeostatic modulator at the intersection of stress endocrinology and neuroinflammation. Furthermore, the impact of ERBB4 dysregulation and its mutations on neurodegenerative diseases has been presented, focusing on potential mechanisms of oxidative stress, synaptic dysfunction, and neuronal apoptosis. Taken together, ERBB4 represents an important molecular interface between stress signaling and neurodegenerative pathology. Understanding the regulation of ERBB4 in the HPA axis has provided new insights into the mechanisms underlying neurodegenerative diseases and could identify novel therapeutic targets for stress-associated neurological disorders.

## 1. Introduction

The hypothalamic–pituitary–adrenal (HPA) axis is a communication system among the hypothalamus, pituitary gland, and adrenal cortex that is essential for regulating chronic stress responses and maintaining homeostasis, mood, energy, metabolism, and immune functions [1,2]. The HPA axis is activated by various stress signals and provides a long-term stress response (10 s) after the initial activation of the sympathetic nervous system. The hypothalamus can release the stress response regulator corticotropin-releasing hormone (CRH) and stimulate the anterior pituitary gland, resulting in adrenocorticotropic hormone (ACTH) release into the bloodstream. After reaching the adrenal gland, ACTH binds to the adrenal cortex and releases cortisol into the bloodstream [1,3,4,5]. Glucocorticoid receptors (GR) and mineralocorticoid receptors (MR) in the hypothalamus and hippocampus detect cortisol. When cortisol levels reach the physiological threshold, they inhibit CRH production, terminating prolonged HPA axis activity through a negative feedback loop [6,7]. Dysregulation and impaired functions of the HPA axis, including enhanced cortisol release, were reported in anxiety-associated diseases, such as depressive disorder (MDD) and borderline personality disorder (BPD) [8,9]. Conversely, an exaggerated negative feedback response driven by increased GR binding and low baseline cortisol levels increased the risk of post-traumatic stress disorder (PTSD) [10]. HPA dysfunctions were also associated with memory impairment through glucocorticoid-related mechanisms and impaired brain structures, including the hippocampus and prefrontal cortex [8,9,11].

Receptor tyrosine-protein kinase ERBB4 is a cell surface receptor for neuregulin 1–4 (NRG1, NRG2, NRG3, NRG4) and other EGF-like growth factors. ERBB4 and other ERBB receptors (ERBB1, ERBB2, ERBB3) are activated by extracellular growth factor ligands, including EGF, transforming growth factor alpha, amphiregulin, betacellulin, epiregulin, and heparin-binding EGF-like growth factor [12,13]. Activation of ERBB4 regulates different cellular processes, including cell proliferation, differentiation, survival, migration, and apoptosis [14,15,16]. The NRG1/ErbB4 signaling pathway was found to significantly influence the neurological response to stress and protect against stress-related behaviors, including depression, as NRG1 administration improved depression-like symptoms in mice [17,18]. ERBB4 dysfunction was suggested to impact the onset of frontotemporal dementia–amyotrophic lateral sclerosis (FTD-ALS), Alzheimer’s disease (AD), and schizophrenia by reducing neural resilience to stress [19,20,21,22,23].

The NRG1–ERBB4 signaling pathway potentially interacts with the HPA axis and directly influences stress responses [24,25]. ERBB4 was found to be expressed in both the adrenal cortex and hypothalamus, suggesting a role in the regulation of HPA axis functions and stress responses [25,26,27]. Dysregulation of ERBB4 signaling was shown to influence HPA dysfunction and stress-related diseases, especially through the ERBB4-NRGH1 pathway. Mice with Nrg1 knockout were subjected to repeated stress, revealing reduced plasma corticosterone levels compared with normal NRG1 mice, demonstrating altered NRG1 dysfunction in the activity of the HPA axis [28,29].

As mentioned above, dysfunction of the HPA axis was closely associated with hormonal dysregulation, resulting in abnormal stress responses [28,29]. Since abnormal stress was suggested to affect several mental diseases and neurodegenerative diseases, such as AD, ALS, and PD, the involvement of ERBB4 in these diseases through its potential influence on the HPA axis was discussed. In this review, the involvement of ERBB4 as a critical, stress-responsive molecular interface operating at the intersection of neuroendocrine (HPA axis) regulation and neurodegenerative vulnerability was discussed. Recently, aberrant ERBB4 expression in excitatory neurons was suggested to be a marker for neurodegeneration, especially for AD. Rather than treating ERBB4 merely as a localized growth factor receptor, the effects of age- and pathology-dependent disruptions of ERBB4-NRG1 signaling on the induction of a feed-forward neuroendocrine/neuroinflammatory cascade were presented, including accelerated disease progression across distinct neurodegenerative etiologies.

## 2. ERBB4 and NRG1 Structure, Functions and Pathways

**Figure 1:**
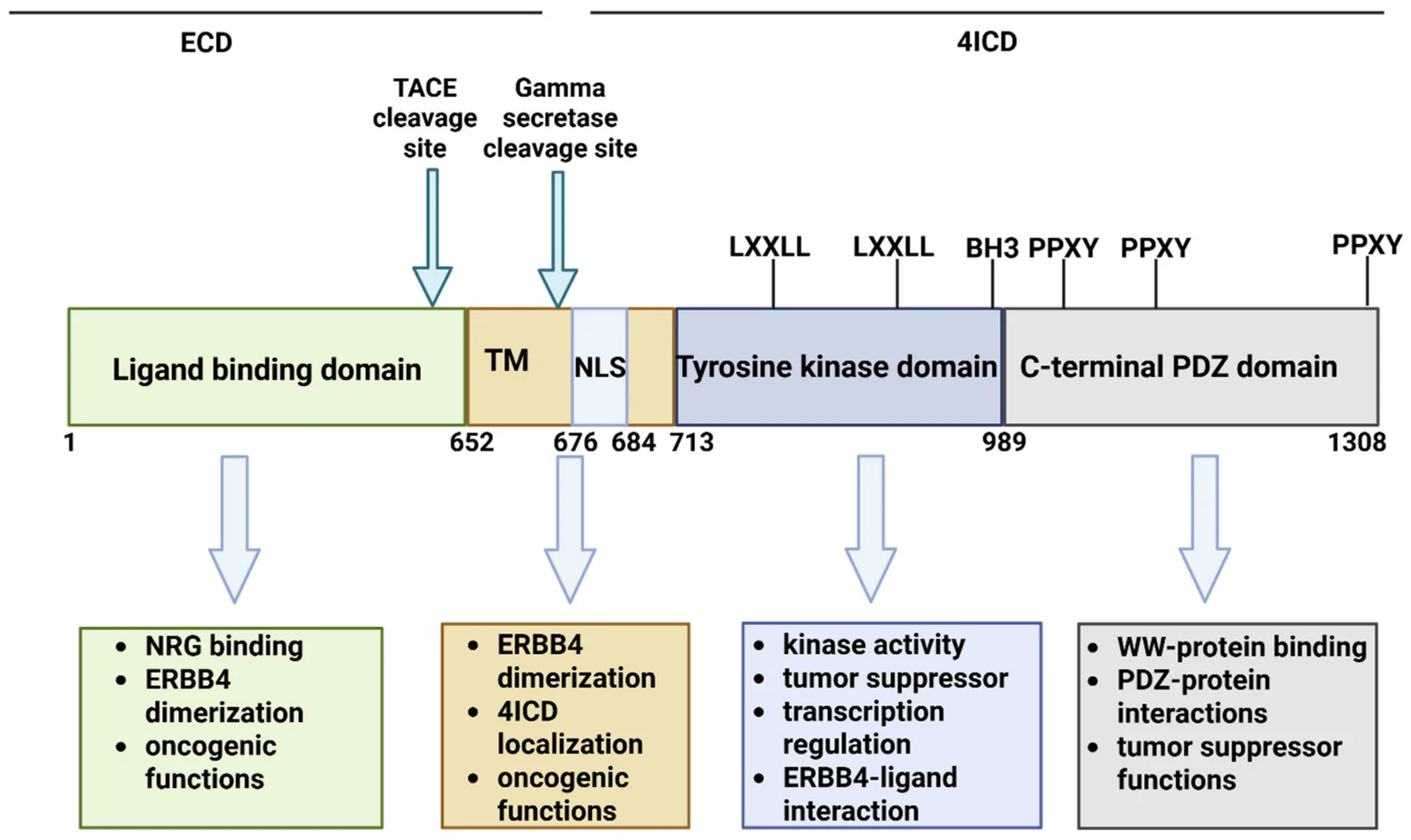
ERBB4 protein with different domains and functions [33]. Created in BioRender. An, S. S. A. (2026) https://BioRender.com/tu8avcm.

ErbB4 is a type I transmembrane protein with a single pass-through TM region, along with four distinct functional domains [12,13,30]. The extracellular domain (ECD) of ERBB4 at the N-terminus is responsible for ligand binding and receptor dimerization. The ECD contains four subdomains (I–IV), of which I and II are involved in ligand binding to neuregulin molecules, betacellulin (BTC), epiregulin (EREG), and heparin-binding EGF-like growth factor (HBEGF) among the EGF family members. The ECD region was reported to be involved in oncogenic functions. Ligand binding at the ECD induced ERBB4 homodimerization or heterodimerization with other receptors, including HER2, playing a significant role in signal transduction, and this domain contained a binding site for tumor necrosis factor-α-converting enzyme (TACE) and ADAM17 [30,31]. The TM domain of the ERBB4 protein was responsible for ligand dimerization and contained a γ-secretase cleavage site [30,32,33,34]. Furthermore, ERBB4 contained a cytoplasmic tyrosine kinase domain responsible for catalytic activity, including ligand phosphorylation and autophosphorylation. Two LXXLL motifs were reported in this region between residues 780–784 and 864–868, which were responsible for the tumor suppressor activity and interactions between ERBB4 and steroid hormone receptors. The tyrosine kinase domain contained a BH3 motif (residues 985–992), which was involved in tumor suppression by controlling interactions between Bcl proteins and ERBB4 [29,35]. The fourth domain of ERBB4 was the C-terminal PDZ domain-binding motif, where three proline-rich (PPxY) motifs were identified. These PPxY motifs bound to WW domain-containing proteins, including different tumor suppressor proteins. This domain interacted with PDZ domain-containing proteins, such as PSD-95/SAP90, PSD-93/chapsyn-110, and SAP102, thereby activating ERBB4 [33,35]. Figure 1 summarizes the ERBB4 structure and its functions [33,36,37].

NRG1 was a membrane glycoprotein with multiple protein isoforms (at least 30). All NRG1 isoforms presented several key domains, such as an immunoglobulin-like domain, an EGF-like domain, a TM domain, and a cytoplasmic tail [29,38]. NRG1 was responsible for cell proliferation, differentiation, survival, and migration. Interestingly, NRG1 was also involved in neural and cardiac development and homeostasis [38,39]. In the brain, NRG1 was associated with axonal myelination, synaptic plasticity, and neuroprotection [40].

The ERBB4-NRG1 interaction resulted in dimerization: either homodimer formation (ERBB4 and ERBB4) or heterodimer formation (between ERBB4 and ERBB1, ERBB2, or ERBB3). Homodimerization activated the ERBB4 tyrosine kinase domain and the autophosphorylation of Tyr1056 in the intracellular domain [41,42,43]. Phosphorylation of the Tyr1056 residue was a molecular switch that induced recruitment of signaling molecules, including the p85 subunit of PI3K, PLCγ2, and Src [44]. The ERBB4-NRG1 interaction activated neuroprotection through the PI3K/Akt pathway. Upon activation, PI3K converted phosphatidylinositol 4,5-bisphosphate (PI4,5P) into phosphatidylinositol 3,4,5-trisphosphate (PIP_3), which subsequently recruited and phosphorylated the Akt pathway. This cascade exerted anti-apoptotic effects by inhibiting caspase-9 expression and modulating the Bcl-2 family proteins [45,46,47,48]. Furthermore, NRG1–ERBB4 signaling mitigated inflammatory damage by inhibiting the JAK2/STAT3-MMP3 and NF-kappaB pathways [38].

In addition to neuroprotection, ERBB4 influenced broader cellular and systemic functions, including cell proliferation and development by interacting with NRG molecules through the MAPK/ERK signaling pathway [49]. Furthermore, ERBB4 controlled the extracellular dopamine pathways through the p38-MAPK pathway [50] and lung development through STAT signaling [51]. In neuronal development, ERBB4 interacted with NRG3 by inducing the development of excitatory synapses of inhibitory GABAergic neurons. High expressions of NRG3 and ERBB4 determined synaptogenesis in the second half of development, especially the formation of excitatory synapses [52]. While PI3K/Akt and MAPK/ERK cascades were ubiquitous in survival and mitogenic pathways across epithelial and cardiac tissues, their downstream biological role of ERBB4 in the mature brain was shown to be structurally distinct: (1) In post-mitotic cortical and hippocampal neurons, ERBB4-mediated PI3K/Akt signaling did not drive cell division; instead, it regulated local translation, stabilized postsynaptic density protein 95 (PSD-95), and maintained synaptic E/I balance [45,46]. (2) Downstream of ERBB4, suppression of the JAK2/STAT3-MMP3 and NF-kappaB pathways functioned as an anti-inflammatory circuit breaker in central microglia and astrocytes [28,47,48]. This was found to contrast sharply with peripheral tissues, oncogenic states and AD conditions, where STAT3 activation was predominantly reported to induce proliferation or invasive metastasis [49,50,51,52,53,54,55].

**Figure 2:**
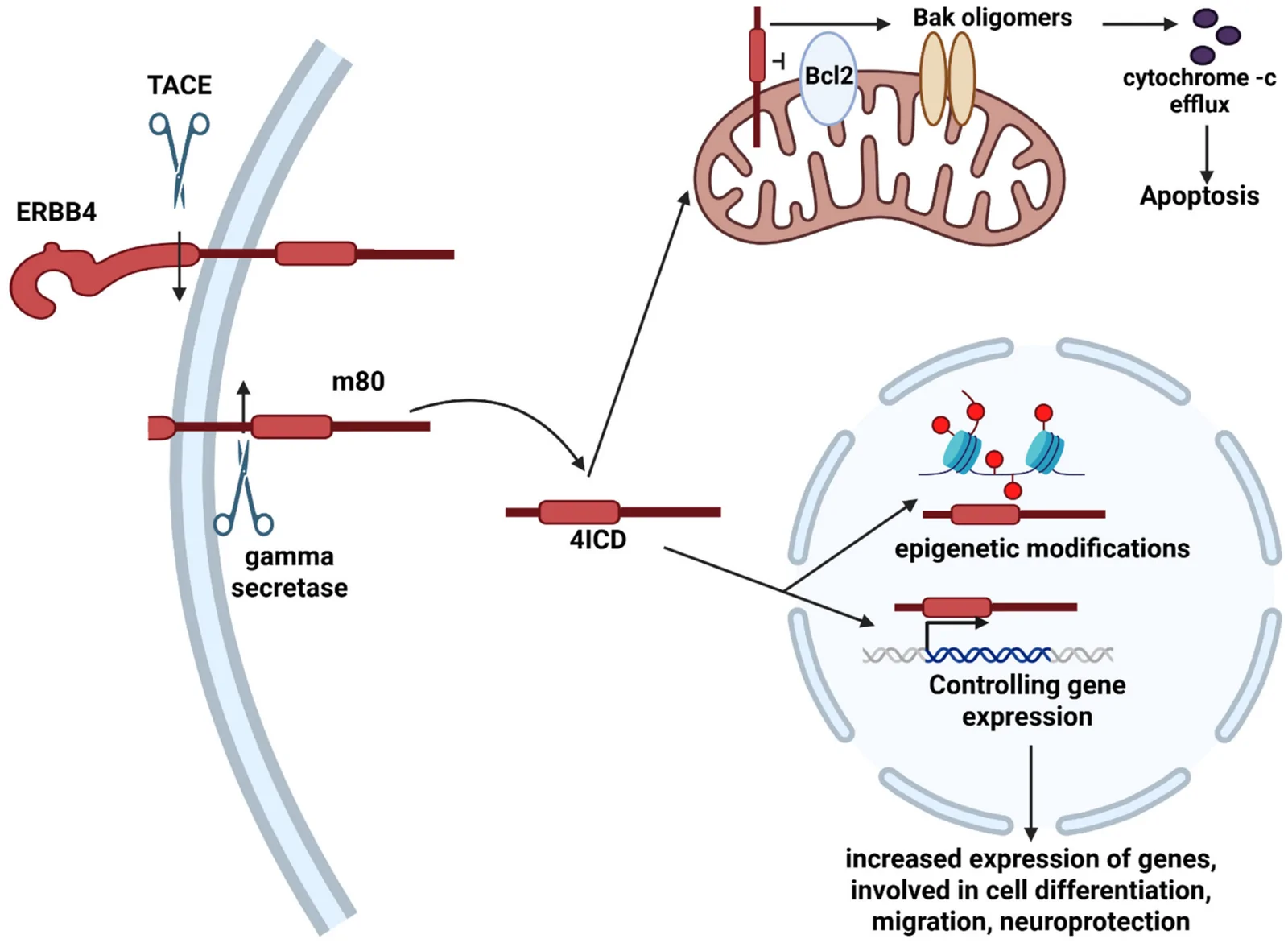
Role of ERBB4-4ICD inside the cells. Created in BioRender. An, S. S. A. (2026). https://BioRender.com/9k92ly7.

ERBB4-4ICD was critical in the regulation of gene expression in the brain. The release of 4ICD from protease-sensitive ErbB4 isoforms (JM-a, JM-d) was carried out in two steps: the primary cleavage was processed by TACE, releasing the extracellular domain and leaving a membrane-bound intracellular domain (m80). The m80 fragment was cleaved by γ-secretase (secondary cleavage), which released the soluble 4ICD into the cytoplasm (Figure 2) [14,56,57]. The functional destination of 4ICD controlled the divergent cellular outcomes, presenting a physiological dichotomy that depended heavily on cellular stress levels and model context. From the cytoplasm, 4ICD was translocated to the nucleus. By interacting with nuclear proteins and chromatin, 4ICD was processed through post-translational pathways, including SUMOylation [58], ubiquitination, and degradation [59]. This 4ICD regulation in the nucleus was essential for neuronal gene expression and regulation, including migration, differentiation, synaptic plasticity, and oligodendrocyte maturation [60,61,62]. 4ICD was reported to control the transcription of different genes in brain development and functions, including genes associated with semaphorin signaling (PAK1, ROCK, and RhoA), actin cytoskeletal plasticity (including F-actin, PFN, or PI3K), glutamatergic-GABAergic activity-related genes, or dendritic spine (including PFN2, ACCN1) morphogenesis [60,61]. 4ICD was also associated with anti-apoptotic effects, since its inhibition resulted in increased vulnerability to neurotoxic stress signals, including Bcl-2 [62]. Beyond the nucleus, the 4ICD was controlling mitochondrial functions by translocating to the mitochondrial membrane, inhibiting Bcl-2, and interacting with BAK oligomers, resulting in cytochrome c efflux and apoptosis [53,63,64].

## 3. ERBB4 in the HPA Axis: Physiological Roles in the Brain

**Figure 3:**
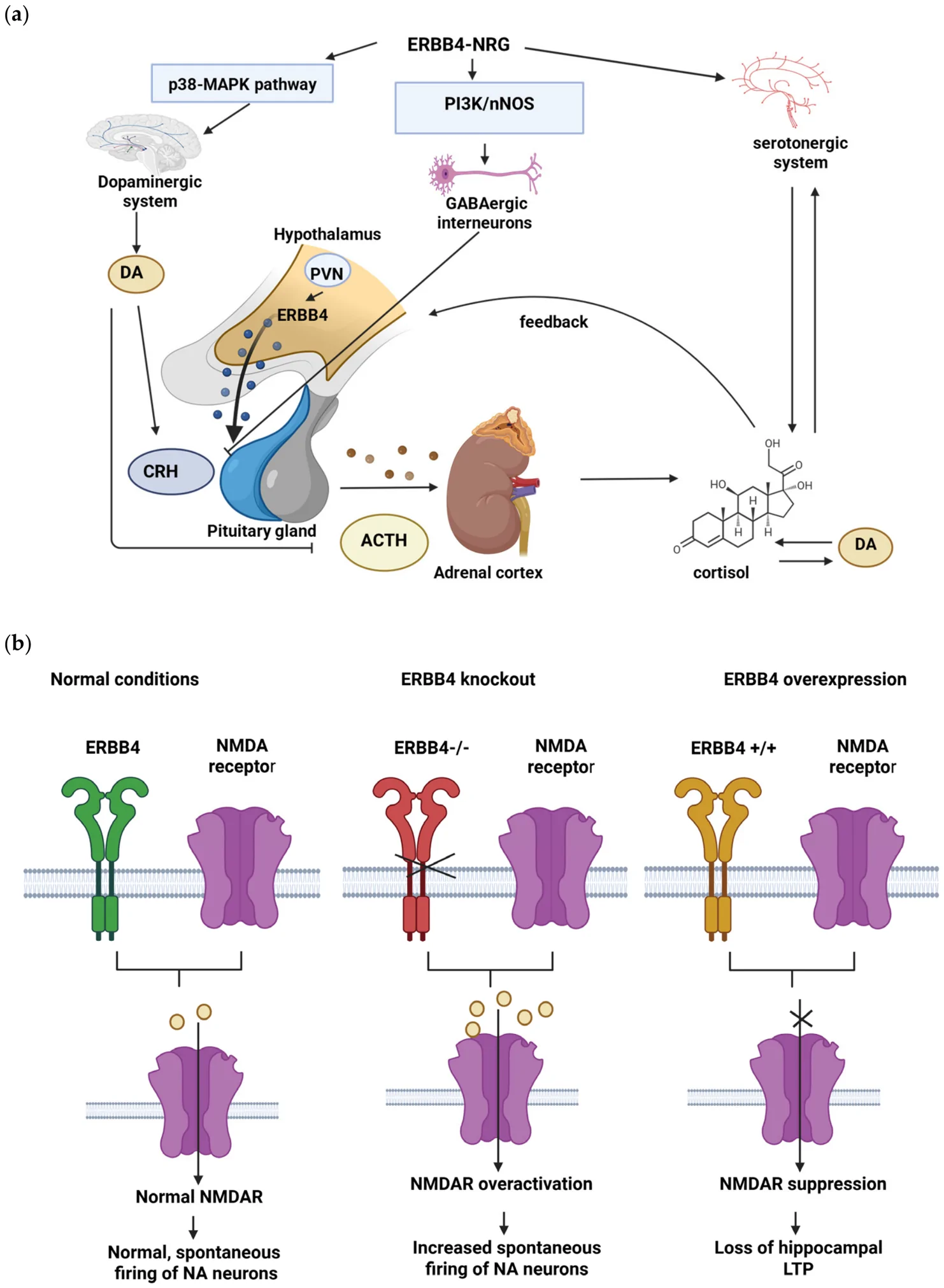
(**a**) Potential effects of ERBB4 on HPA axis association in stress response. (**b**) Role of ERBB4 in noradrenergic neurons. Created in BioRender. An, S. S. A. (2026) https://BioRender.com/klckuy7.

While these core kinase and proteolytic cleavage cascades established ERBB4 signal transduction at the cellular level, understanding its broader physiological role required analyzing how these pathways functioned across neural circuits and neuroendocrine regulatory centers, particularly the HPA axis [64,65]. To understand how ERBB4 regulated the neuroendocrine dynamics, its functions should be divided into five distinct anatomical and signaling nodes of the HPA axis: (1) hypothalamic CRH neurons; (2) pituitary ACTH regulation; (3) adrenal glucocorticoid production; (4) glucocorticoid receptor (GR) signaling crosstalk; and (5) feedback regulation in the hippocampus and prefrontal cortex (PFC) [66,67,68]. Figure 3a illustrates the possible impact of ERBB4 on the HPA axis.

As mentioned above, the HPA axis was a primary neuroendocrine feedback loop involved in stress regulation. ERBB4 was expressed in the serotonergic system, where it influenced the stress response by impacting overall neuroendocrine balance and behavioral responses [43,68]. It played a critical role in neuronal development, including axonal arborization in the forebrain [69]. ERBB4 dysfunction disrupted serotonergic connectivity, increased the excitability of 5-HT neurons, and impaired memory formation [70,71]. For instance, ERBB4 deficiency in dorsal raphe nucleus (DRN) 5-HT neurons resulted in anxiety-like behaviors and impaired fear extinction in mice [71]. Furthermore, an ERBB4 imbalance altered the reciprocal relationship between serotonin and glucocorticoids [69].

ERBB4 was expressed in the PVN of the hypothalamus and local GABAergic interneurons. NRG1/ERBB4 signaling promoted local GABA release, acting as a crucial central brake that restrained CRH neuronal hyperexcitation. Furthermore, hypothalamic ERBB4 expression was regulated by estrogen receptor-beta (ER-beta) signaling. Depletion of ER-beta reduced ERBB4 levels in the PVN, leading to unrestrained CRH release and chronic HPA axis hyperactivity [17,26,68,72,73,74]. NRG1 influenced the size and number of excitatory synapses, thereby controlling their formation on GABAergic interneurons [69]. Mice with ERBB4 knockout presented fewer interneurons and reduced GABA release, resulting in behavioral deficits that were reversed by restoring adult ERBB4-NRG1 signaling [75,76]. Additionally, ERBB4-NRG1 signaling controlled neuronal nitric oxide synthase (nNOS) activation and GABA release through PI3K pathways. Deletion of nNOS in ERBB4-positive neurons disturbed GABAergic transmission, inducing schizophrenia-like behaviors, such as hyperactivity, abnormal working memory, and impaired social interactions [76]. Ultimately, deficient ERBB4 and nNOS functions impaired GABAergic transmission, increasing the excitation/inhibition (E/I) ratio and disrupting pyramidal neuron activity [77,78].

Furthermore, ERBB4 modulated dopamine (DA) levels and dopaminergic neuron function by regulating extracellular DA through the p38-MAPK pathway [78]. In SH-SY5Y cells, inhibiting ERBB4 increased the ratio of phosphorylated to total p38, elevated extracellular DA levels, and altered dopaminergic axonal projections [51]. Conversely, studies on Lund Human Mesencephalic (LUHMES) cells revealed that NRG/ERBB signaling increased DA levels by reducing dopamine transporter-dependent uptake [78,79]. Activation of ERBB receptors also induced DA release in the hippocampus, where NRG-1-mediated long-term potentiation (LTP) and depotentiation depended on D4 dopamine receptors to reduce synaptic AMPA-type glutamate receptors [80]. In ErbB4-deficient mice, steady-state DA levels were reduced in the medial prefrontal cortex, hippocampus, and nucleus accumbens, but they were elevated in the dorsal striatum [80,81]. Taken together, ERBB4 function was critical in modulating DA and GABAergic systems alongside the HPA axis, forming a complex regulatory network that could maintain neuroendocrine balance and behavioral stability [37,82].

At the systemic level, ERBB4 maintained metabolic homeostasis within the hypothalamus and regulated stress responses in the bed nucleus of the stria terminalis (BNST) [17,41,82]. Inhibiting NRG1–ERBB4 signaling with antagonists in the mouse BNST induced anxiety-like behavior by impairing presynaptic GABA release [17,78]. Infusion of a GABA-A receptor antagonist resulted in similar behavioral outcomes without compounding the effects of ERBB4 blockade, supporting the involvement of a shared pathway [17,41,83]. Beyond neurotransmitter regulation, ERBB4 protected the cells against oxidative and endoplasmic reticulum (ER) stress [50]. For example, ERBB4 overexpression in mesenchymal stem cells enhanced tolerance to the ROS hydrogen peroxide [50], while NRG1 treatment in ERBB4-expressing PC12 cells mitigated oxidative stress through the PI3K-PKB/Akt pathway [84].

Beyond hypothalamic drivers, ERBB4 expression in the anterior pituitary modulated corticotrope responsiveness [85]. Disruption of central and peripheral ERBB4 signaling increased the circulating ACTH levels under stress, altering the dynamic range of pituitary endocrine output [1,2,3,4,5,85,86,87,88]. In addition, ERBB4 was expressed directly within the adrenal cortex. NRG1 or ERBB4 dysfunction altered the systemic corticosterone/cortisol surges after stress, supporting the role of ERBB4 in maintaining physiological steroidogenesis at the adrenal level [18]. ERBB4 was also involved in GR signaling crosstalk: the conserved LXXLL motifs of ERBB4 mediated the direct physical and functional interactions with nuclear steroid hormone receptors and modulated downstream GR signaling pathways in response to elevated circulating glucocorticoids [6,7,89,90,91,92,93]. Finally, ERBB4 was involved in feedback regulation in the hippocampus and prefrontal cortex, where ERBB4 was expressed in PV+ interneurons and induced the E/I balance and LTP, which was essential for GR-mediated negative feedback. Impaired ERBB4 induced the pro-inflammatory cascade and enhanced the feed-forward failure of HPA negative feedback [91].

ERBB4 was also shown to be expressed in noradrenergic neurons (NA), where it was suggested to regulate the mechanism of spontaneous neuronal firing. ERBB4 modulated the N-methyl-D-aspartate (NMDA) receptor activity and was involved in stabilizing the excitability of noradrenergic circuits [27,66,80]. Loss or knockout of ERBB4 resulted in an increased rate of spontaneous firing of noradrenergic neurons and increased levels of norepinephrine [67] (Figure 3b), which helped to maintain cognitive functions in an AD animal model. ERBB4 overactivation suppressed NMDAR activation, which resulted in reduced hippocampal LTP [55,94].

Furthermore, ERBB4 did not function only as a systemic correlate of cortisol/corticosterone, but also acted as a functional modifier across additional anatomical nodes of the HPA axis [48,94,95,96,97,98,99].

## 4. ERBB4 Dysfunctions in Neurodegeneration and the Involvement of the HPA Axis

### 4.1. ERBB4 Mutations and Dysfunctions in Neurodegenerative Diseases

#### 4.1.1. ERBB4 and Alzheimer’s Disease (AD)

In AD patients, ERBB4 expression was elevated across several brain regions, including the corticomedial amygdala, human basal forebrain, and superior frontal gyrus [27]. Similarly, in the hippocampus of both AD patients and APP/PS1 mice, ErbB4 and phospho-ErbB4 immunoreactivities showed higher intensity in the neurons of the CA1–CA2 transitional field compared to controls [27]. Furthermore, ERBB4 levels were increased in the apoptotic hippocampal pyramidal neurons of AD patients, where it co-localized with the Bax apoptotic signal, suggesting its direct involvement in AD-related neuronal loss [65].

In the healthy mature CNS, ErbB4 expression was restricted to PV+ inhibitory interneurons. Recent translational and human studies, however, showed that ERBB4 was upregulated in excitatory neurons (but not in inhibitory) of AD patients/AD mice. Single-nucleus RNA-sequencing analysis revealed that ERBB4 overexpression in early responsive excitatory neurons (EREN) was one of the earliest alterations in AD mice. Interestingly, the increased ERBB4 expression resulted in several abnormalities, including hyperactivity, altered glial pruning, and reactive gliosis, causing cognitive decline in animal models [55].

ERBB4 was also thought to modulate amyloid metabolism, and its inhibition protected against amyloid-beta (Aβ) and synaptic toxicity. Direct interaction was reported between ERBB4 and Aβ, and this interaction interfered with physiological ERBB4-NRG1 signaling and induced several deleterious downstream cascades. Specifically, Aβ-mediated ERBB4 activation was reported to stimulate the JNK pathway, which could be involved in Tau phosphorylation [100,101]. This interaction was also reported to promote neuroinflammation through p38/MAPK pathways [100,101]. Additionally, aberrant ERBB4-NRG1 signaling dysregulated the Akt/mTOR axis, increasing the expression of potassium channel proteins; hyperactivation of this mTOR pathway further elevated total Tau levels and accelerated its pathological phosphorylation [102,103]. ErbB4 overexpression in excitatory pyramidal neurons initiated the downstream mTOR Complex 1 (mTORC1) signaling cascade and the downregulation of genes involved in the functions of voltage-gated potassium channels, resulting in constant excitability of CA1 pyramidal neurons. It also resulted in abnormal microglial and astrocyte activation and excitatory synapse engulfment [55]. The ERBB4 levels correlated positively with plaque severity and negatively with cognitive function, suggesting it as an upstream switch of their converged degenerative pathways [55].

In PV interneurons, ERBB4 deficiency accelerated disease pathology. In the olfactory bulbs of APP/PS1 mice, impaired ERBB4 enhanced CDK5 signaling, which stimulated β-secretase (BACE1) activity and increased Aβ deposition [104]. This impairment was related to a loss of GABAergic inhibition, resulting in neuronal hyperexcitability, excitation/inhibition (E/I) imbalance, and subsequent cognitive dysfunction. In APP mouse models, the number of PV interneurons was significantly reduced in the hippocampal cornu ammonis 1 (CA1) and dentate gyrus (DG) regions [19,80,105]. Levels of proteins essential for GABA synthesis and transport, including GAD65/67 and vesicular GABA amino acid transporter (vGAT), were also reduced. Consequently, hippocampal synaptic transmission and LTP induction were impaired, alongside a reduction in the synaptic proteins PSD-95 and Syn-1. While these mice exhibited severe cognitive decline and epileptic symptoms, exogenous NRG1 administration improved their symptoms [19,80,105]. Erbb4 deletion in AD excitatory neurons protected against abnormal neuronal network activity and synapse loss, reactive gliosis, amyloid plaque deposition, and cognitive deficits.

**Figure 4:**
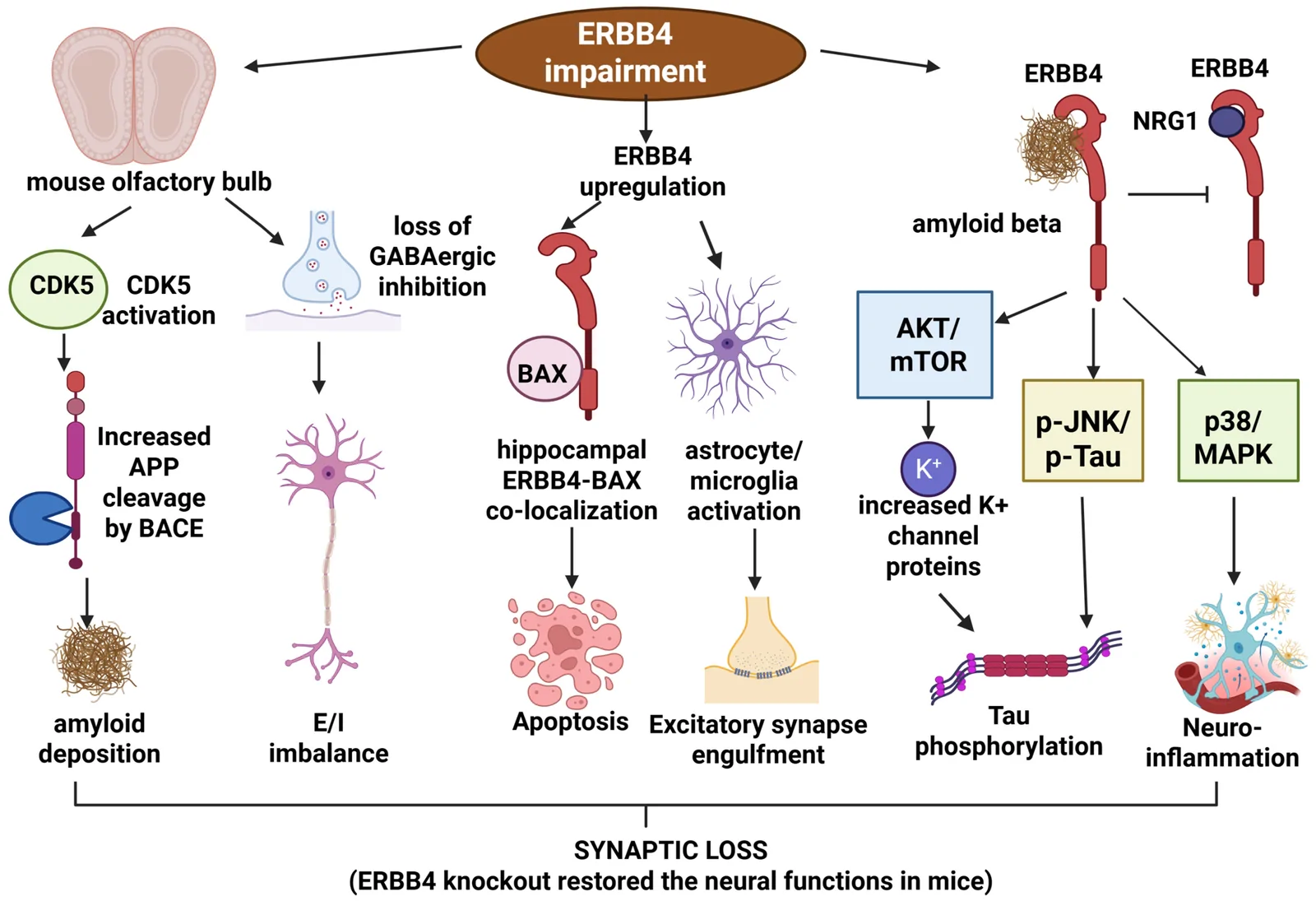
Potential impact of ERBB4 dysfunction in AD [19,27,55,66,100,101,102,103,104,105,106,107,108,109,110,111,112,113]. Created in BioRender. An, S. S. A. (2026) https://BioRender.com/cih1smx.

However, the issue could be that even though cell-line overexpression models (e.g., HEK293/SH-SY5Y) successfully mapped the ERBB4–Aβ binding and downstream kinase cascades, these artificial conditions can induce intracellular signaling responses or force non-physiological protein interactions relative to intact neural circuits, which did not reflect the in vivo pathways. Conversely, while transgenic mouse models (e.g., *APP*/*PS1*) successfully captured circuit-level E/I imbalances, they frequently modeled aggressive, non-physiological Aβ overproduction rather than the decades-long, low-grade neurodegenerative process observed in sporadic human AD [27,55,106,107,108,109,110,111,112,113]. Figure 4 summarizes the possible pathways of ERBB4 dysfunction in AD progression.

#### 4.1.2. ERBB4 and Frontotemporal Dementia/Amyotrophic Lateral Sclerosis Type 19 (ALS19)

ERBB4 was highly expressed in motor neurons and played a crucial role in their function, plasticity, and survival [20]. The NRG1–ERBB4 pathway was highly involved in glia–axon interactions, myelination, neuronal migration, and synaptogenesis. This pathway could regulate the C-bouton synapses, which are unique cholinergic terminals on spinal motor neurons. In these synapses, NRG1 was concentrated postsynaptically within the subsurface cistern of the endoplasmic reticulum, while ErbB2 and ErbB4 receptors resided in the presynaptic compartment, suggesting a retrograde signaling mechanism. Consequently, impaired ERBB4 and NRG1 signaling was associated with abnormal microglial activation and neuroinflammation [20]. In sporadic ALS patients, motor neurons in the anterior horns of the spinal cord showed reduced ERBB4 immunoreactivity, which was frequently described and correlated with the severity of motor neuron degeneration and the cytoplasmic mislocalization of TDP-43. Furthermore, abnormal nuclear or nucleolar accumulation of ERBB4 and spheroid formation were observed in ALS pathology [114,115,116].

**Figure 5:**
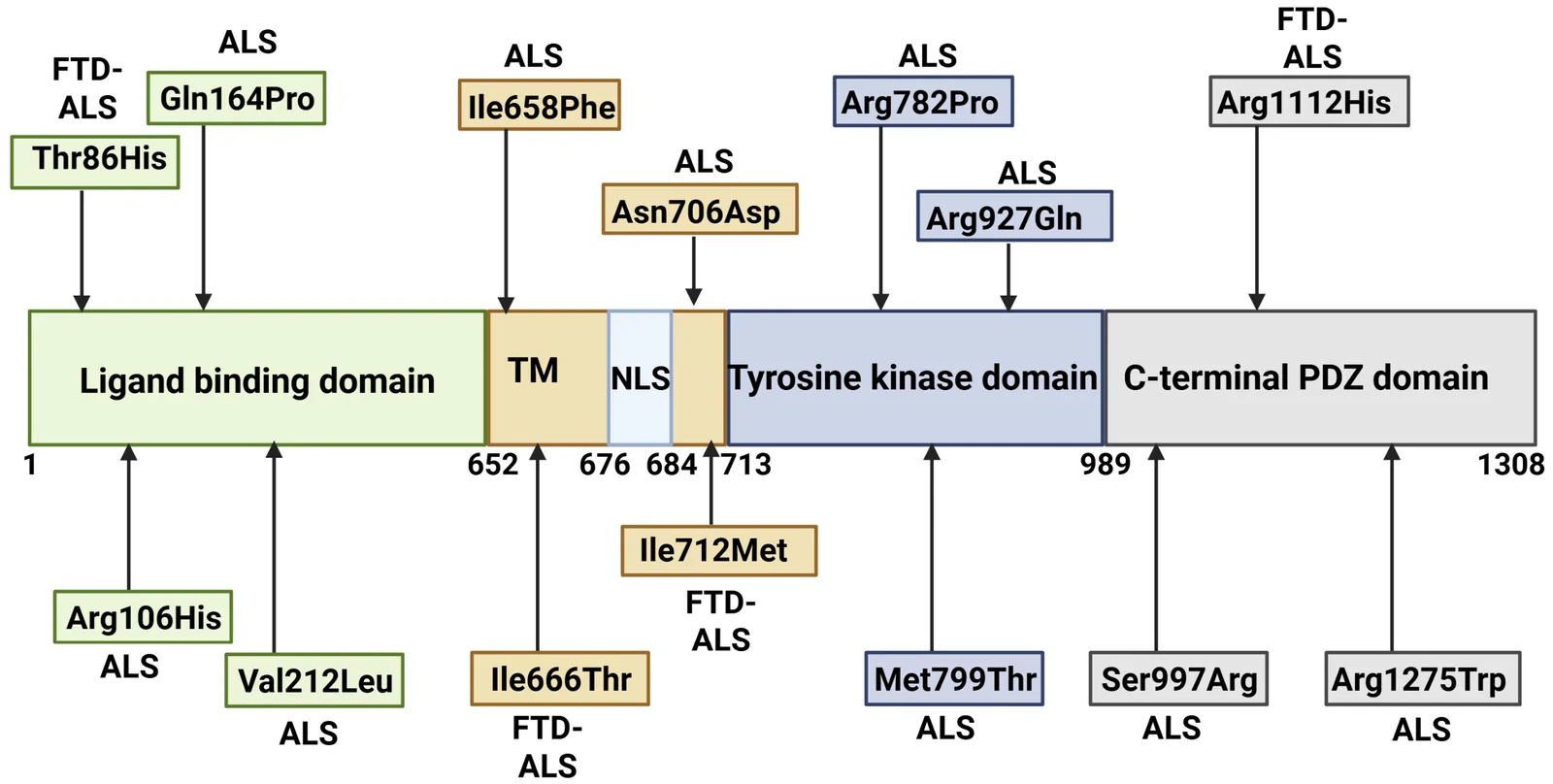
Location of ALS- and FTD-ALS-associated mutations in the ERBB4 protein. Created in BioRender. An, S. S. A. (2026) https://BioRender.com/5dke5nm.

Published genetic studies revealed that rare ERBB4 variants were estimated to account for less than 1% of total ALS cases globally (pooled frequency ~0.83%, and ~0.67–1.36% across specific European and East Asian cohorts), making them a rare genetic cause within familial ALS (fALS) and ALS-FTD spectrum disorders [116,117,118,119,120,121,122,123,124,125,126] (Table 1, Figure 5). Functional analyses suggested that pathogenic *ERBB4* mutations acted through loss-of-function (LoF) mechanisms, severely compromising ligand-induced autophosphorylation and downstream survival signaling, the PI3K/Akt and EK/ERK1/2 pathways [116,120]. Structurally, these variants generally clustered into two functional regions: (1) extracellular ectodomain variants (N-terminal cysteine-rich domains), including Arg106His, Gln164Pro, and Val212Leu, were identified in the conserved furin-like cysteine-rich domains, required for NRG1 binding, receptor dimerization, and crosstalk with canonical Wnt-beta-catenin signaling [118]. These alterations disrupted ligand recognition and extracellular structural transitions [116,117,118,119,120,121,122,123,124,125,126]. (2) Intracellular tyrosine kinase domain (TKD) variants: mutations, including Asn706Asp, Ile712Met, and Arg927Gln, were suggested to directly alter the catalytic ATP-binding pocket or phosphotransferase core, abolishing NRG1-stimulated kinase activation [116,117,118,119,120,121,122,123,124,125].

A critical question in ALS19 pathology should be: why did specific *ERBB4* variants manifest as isolated motor deficits while others resulted in cognitive–behavioral impairment? ERBB4 variants, including Arg927Gln and Asn706Asp, presented with classic spinal- or bulbar-onset motor neuron degenerations characterized by progressive limb weakness, fasciculations, and muscle atrophy, but no cognitive or behavioral dysfunction [116,122]. Conversely, variants including Ile712Met and Tyr86His were associated with frontotemporal cortical atrophy, executive dysfunction, behavioral modifications, and language impairment alongside motor neuron loss [116,117,118,119,120,121,122,123,124,125,126]. This phenotypic divergence suggested that specific variants differentially affected cell-type-specific co-receptors or downstream interactors in cortical interneurons and spinal motor neurons [116,117,118,119,120,121,122]. Furthermore, *ERBB4* variants also interacted with other genetic risk factors; for instance, co-inheritance of ERBB4 (Tyr86His) and progranulin (*GRN* Pro451Leu) in an FTD patient resulted in both behavioral and motor dysfunction and was associated with accelerated cortical neurodegeneration [124].

A significant limitation of studies on ERBB4 and ALS or FTD-ALS could be the small sample size of patients carrying specific rare ERBB4 variants, which hindered the establishment of robust genotype–phenotype correlations [118,121,124]. Since the involvement of ERBB4 in ALS/FTD or pure ALS was discovered relatively recently, the mutations in this gene were overlooked; the majority of variants in ERBB4 were categorized as variants of unclear significance (VUS) [118,121,124]. Another issue was the clinical heterogeneity of mutations, suggesting the presence of putative genetic or environmental factors [109,116]. Additionally, functional studies of ERBB4 mutations were limited. While ERBB4 Ile712Met and p.Arg927Gln were verified to reduce receptor autophosphorylation upon NRG1 stimulation, these in vitro findings did not fully replicate the complex, cell-type-specific environment of the human brain. Moreover, these immortalized lines limited the complex architecture of human motor neurons, including extended axonal transport systems and localized microglial/astrocyte crosstalk [121,122].

#### 4.1.3. ERBB4 and Parkinson’s Disease

ERBB4 was reported to be highly expressed in midbrain DA neurons and regulated dopamine levels, cellular integrity, and survival [78]. Elevated levels of both ERBB4 and NRG1 in midbrain networks provided vital trophic support. Post-mortem tissue analysis revealed that ERBB4-positive DA neurons had significantly higher survival rates and better preservation of melanized cell bodies compared with ERBB4-negative cells, underscoring its neuroprotective role [127].

A primary mechanism through which ERBB4 maintained DA neuron survival was the suppression of ferroptosis (iron-dependent programmed cell death). Specifically, the ERBB4-NRG3 pathway coordinated the crucial neuroglial interactions within the substantia nigra pars compacta (SNpc), preventing the D-galactose-induced neuronal ferroptosis through the CCNE1-PARP16 signaling axis [128,129]. Additionally, ERBB4 signaling interaction provided robust defense against external environmental neurotoxins through the ERBB4-neuregulin-1β1 (Nrg1β1) interaction, including 1-methyl-4-phenyl-1, 2,3,6-tetrahydropyridine (MPTP) and 6-hydroxydopamine (6-OHDA) [130]. While these functional studies demonstrated the robust anti-apoptotic capacity of ERBB4 activation, they failed to model the progressive, age-dependent accumulation of misfolded α-synuclein oligomers, which were characteristic of idiopathic PD. Evaluating ERBB4 protection in endogenous, slow-progressing α-synuclein preformed fibril (PFF) or knock-in models was needed to establish true translational relevance [127,128,129,130].

### 4.2. ERBB4 and Psychiatric Diseases

While schizophrenia was defined primarily as a neurodevelopmental disorder, genetic models targeting ERBB4/nNOS in PV+ interneurons provided the foundational circuit-level evidence that ERBB4 loss induced cortical E/I imbalance and stress vulnerability, which were also suggested to affect neurodegenerative conditions, including AD and ALS [131,132,133,134,135,136,137,138,139].

Variants within the *ERBB4* gene, particularly intronic and splice-site alterations, were genetic risk factors for schizophrenia [124,125]. In a Jordanian Arab population, a strong association was identified between the rs839523 intronic variant and schizophrenia [131]. Similarly, a Chinese cohort study indicated that the synonymous variant Val1065Val, alongside the rs3748962 (CT and CC) genotypes, was associated with increased disease susceptibility [132]. Furthermore, the variants rs7598440, rs839523, and rs707284 cluster near exon 3 and form a high-risk haplotype for schizophrenia [134]. Among these, the rs7598440 variant significantly modulated the onset of psychotic experiences (PEs) under acute stress, while its risk-associated A allele correlated with elevated cortical GABA levels [67,134]. At the transcript level, splice-site variants favoring the expressions of exon 16 (JM-a) and exon 26 (CYT-1) isoforms were elevated in the dorsolateral prefrontal cortex (DLPFC) of schizophrenia patients [133]. This altered *ERBB4* splicing selectively affected PV-positive interneurons, inducing the downregulation of PV expression and blunting interneuron activity [133]. Consequently, both NRG1 and ERBB4 expression profiles were elevated in post-mortem brains and patient-derived induced pluripotent stem cells (iPSCs), pointing to a compensatory or dysregulated feedback mechanism [133,134,135].

Since the NRG1/ERBB4 axis participates in neural development and synaptic plasticity, schizophrenia-associated genetic variants impair GABAergic signaling and DA homeostasis [135,136]. Conditional *ErbB4* knockout mice presented severe GABAergic dysregulation within sensorimotor cortical–lateral striatal networks, characterized by abolition of *GAD1* mRNA expression [137,138]. Furthermore, *ErbB4* KO mice mimicked the classic dopaminergic imbalance seen in schizophrenia patients, showing marked DA reductions in the prefrontal cortex, hippocampus, and nucleus accumbens alongside elevated DA levels in the dorsal striatum [81,82]. *ErbB4* KO mice displayed hallmark schizophrenia-like symptoms, including novelty-induced hyperactivity in open-field environments, elevated anxiety, and severe cognitive, social, and motivational impairments [36,81,82,139].

While *ErbB4* deletion or pharmacological inhibition in adult mice disrupted brain circuit wiring and behavior, it did not alter total neuron numbers or gross morphology [75]. Crucially, restoring ERBB4-NRG1 signaling in adult mice reversed the behavioral impairments successfully and normalized synaptic transmission, suggesting this pathway as a promising therapeutic target [75].

### 4.3. ERBB4 as a Stress-Responsive Molecular Interface Regulating Neuroendocrine Balance, Synaptic Stability, and Neurodegenerative Vulnerability

The HPA axis was suggested to be the primary neuroendocrine system governing physiological stress responses. Emerging evidence suggested that ERBB4 was not a primary initiator, but could be a critical homeostatic checkpoint at the intersection of stress endocrinology, synaptic plasticity, and neuroinflammatory regulation. Rather than acting as a simple upstream cause or downstream effector, ERBB4 could function within a bidirectional feed-forward framework: pre-existing endocrine dysregulation downregulated ERBB4 signaling, while the loss of ERBB4-mediated inhibition subsequently disinhibited neuroinflammatory and HPA responses, thereby accelerating disease trajectories [3,85,86,87].

**Figure 6:**
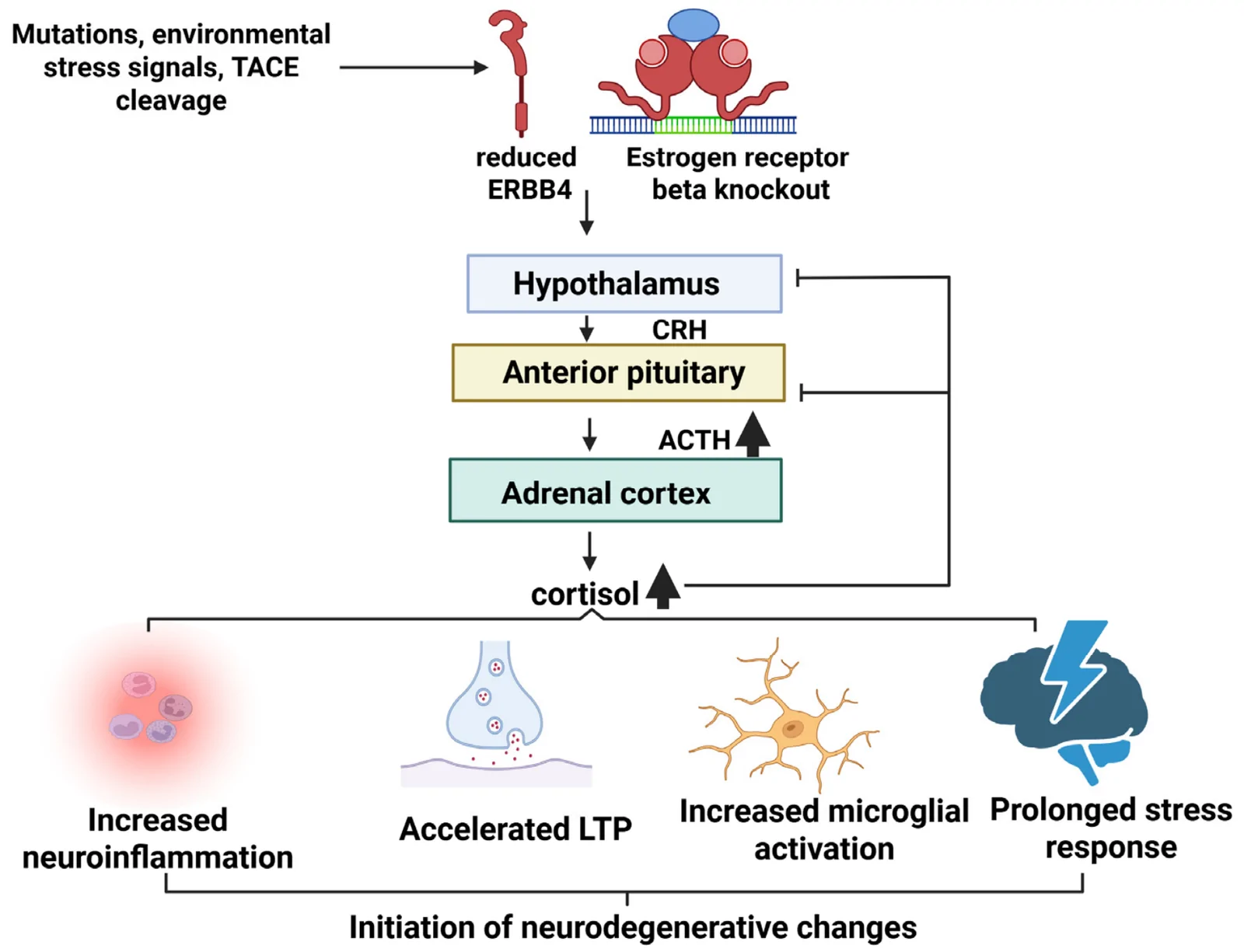
Potential impact of ERBB4 (and estrogen receptor beta) impairment in neurodegeneration through the HPA axis. Created in BioRender. An, S. S. A. (2026) https://BioRender.com/umbndhx.

Hypothalamic ERBB4 functionality was tightly linked to nuclear receptor signaling, particularly estrogen receptor-beta (ER-beta). ER-beta activity maintained baseline ERBB4 expression in the hypothalamus [88]. Mice with ER-beta (Esr2-/-) knockout presented reduced ERBB4 expression in the hypothalamus and fewer ERBB4-positive cells in the paraventricular nucleus (PVN). The lower ERBB4 levels were associated with elevated ACTH and corticosterone levels, resulting in a prolonged HPA response. Excessive glucocorticoid levels damaged the hippocampus by impairing LTP and accelerated brain aging and cognitive decline [88,89,90,91]. Additionally, a decrease in ERBB4 expression impaired secondary neuroprotective mechanisms, including the regulation of oxytocin neurons. This process increased brain vulnerability to chronic stress [26,93]. Beyond baseline endocrine tuning, NRG1–ERBB4 signaling served as an indispensable central brake on neuroinflammation. Under homeostatic conditions, ERBB4 activation suppressed pro-inflammatory cascades in central microglia and peripheral immune cells [37,47,96] by repressing the TLR4–NF-kappaB–NLRP3 inflammasome axis [97,98]. Because chronic stress-induced glucocorticoid secretion promoted central neuroinflammation [3,94,99], and elevated pro-inflammatory cytokines (e.g., IL-1beta, IL-6) activated the HPA axis [94,99], ERBB4 suppressed this destructive neuroimmune–endocrine loop (Figure 6).

The interplay between ERBB4 dysfunction and HPA axis hyperactivity created a permissive feed-forward loop that could accelerate brain aging and modulate vulnerability to neurodegenerative pathologies, including AD, FTD-ALS, and PD [3,85,86,87]. AD clinical progression was strongly correlated with hypercortisolemia [106,107]. Aβ oligomers stimulated glucocorticoid release, while sustained glucocorticoid receptor activation reciprocally enhanced APP processing and Tau phosphorylation [108]. In rodent models, loss of NRG1–ERBB4 signaling removed the anti-inflammatory brake on microglial activation and oxidative stress [3,111,112,113,114], accelerating glucocorticoid- and neuroinflammation-induced synaptic clearance [140].

ALS and FTD-ALS were found to exhibit non-cell-autonomous neuroendocrine involvement. Animal models highlighted a potential modulating role for HPA axis tone in disease progression, but further studies are needed on ERBB4 and the HPA axis in humans [141,142]. Stress stimulation in ALS mouse models induced the corticosterone surge that directly correlated with accelerated astrogliosis, microglial activation, and early-onset muscle paralysis [141,142]. However, further verification would be needed regarding the involvement of ERBB4 and the HPA axis in ALS [142,143,144,145].

In PD, ERBB4 impairment was recognized as a potential modifier of dopaminergic vulnerability to systemic stress and HPA dysregulation [146,147]. While clinical studies confirmed baseline hypercortisolemia and elevated ACTH levels in PD patients [146,147,148,149], these associations did not prove a direct causal link involving ERBB4 mutations in PD patients. Chronic stress and HPA axis hyperactivation accelerated dopaminergic neuronal loss in the substantia nigra pars compacta (SNpc) [150]. In transgenic α-synuclein models, exogenous corticosterone accelerated neurodegeneration by promoting pathological α-synuclein phosphorylation at Ser129 [151,152]. ERBB4 impairment deprived the SNpc dopaminergic neurons of crucial anti-inflammatory tone, increasing their vulnerability to α-synuclein toxicity under hypercortisolemic conditions [146,147].

## 5. ERBB4-Related Biomarkers and ERBB4 as Potential Therapeutic Target of Neurodegenerative Diseases

### 5.1. Therapeutic Strategies and Biomarker Potential of ERBB4

While initial post-mortem immunohistochemical analyses reported the marked ERBB4 upregulation co-localizing with apoptotic markers in hippocampal neurons of AD patients, subsequent functional studies demonstrated contradictory outcomes depending on whether full-length or cleaved intracellular fragments were measured [62]. Additionally, circulating ERBB4 fragments showed strong clinical promise as diagnostic and prognostic biomarkers for motor neuron diseases. Specifically, levels of the 55 kDa soluble ectodomain fragment (ecto-ERBB4) were significantly reduced in the cerebrospinal fluid (CSF) of patients with pure ALS (*n* = 20) and FTD-ALS (*n* = 10) compared with age-matched controls [153]. Quantifying ecto-ERBB4 in plasma and CSF could be a minimally invasive strategy to track disease progression, reflecting the localized loss of full-length ERBB4 receptor expression within degenerating anterior horn motor neurons [116,153,154]. However, large-scale multi-center validation should be performed to evaluate whether ERBB4 levels in plasma and CSF could serve as neurodegenerative biomarkers [154,155,156,157,158].

Given that ERBB4 loss-of-function and aberrant receptor processing were suggested to contribute to AD, ALS, and PD progression, restoring ERBB4 signaling or blocking its pathological cleavage was a promising therapeutic strategy [47,101]. Therapeutic interventions that target the NRG1–ERBB4 axis directly were summarized in Table 2. The small-molecule ERBB4 receptor agonist 4-bromo-1-hydroxy-2-naphthoic acid (E4A or C11H7BrO3) revealed robust anti-inflammatory and neuroprotective effects. In APP/PS1 AD models, E4A suppressed the pro-inflammatory TLR4-NF-kappaB-NLRP3 inflammasome cascade and mitigated microglial activation by upregulating DOCK3 and SIRT3 expression [47,101,159]. This targeted activation ultimately reduced Aβ deposition, preserved mitochondrial integrity, and rescued cognitive and synaptic deficits [1,101,128]. Similarly, the small-molecule EF-1 homodimer inducer activated ERBB4 to upregulate downstream PI3K/Akt and MAPK/ERK survival pathways. EF-1 was utilized for its cardioprotective properties; however, it may also be a promising candidate for inducing neuroprotection [160].

Conversely, pan-ERBB tyrosine kinase inhibitors (TKIs) used in oncology (e.g., neratinib, dacomitinib) were analyzed in neurodegeneration with a different objective: to block the secondary, aberrant overactivation of non-neuronal ERBB receptors (such as microglial EGFR/ErbB1) that could drive reactive astrogliosis [161]. Similarly, the multikinase inhibitor masitinib may modulate downstream ERBB-dependent inflammatory signaling; in clinical trials, masitinib successfully slowed functional decline and prolonged survival in ALS patients [161]. In AD models, blocking pathological JNK hyperactivation using the inhibitor AG1478 or ERBB4-targeted siRNA significantly reduced Tau phosphorylation and Bax-mediated apoptosis, highlighting the necessity of precision targeting in these highly heterogeneous diseases [100,145].

Modulating the sequential cleavage of ERBB4 by TACE and γ-secretase offered an alternative approach to control the release of the ERBB4 intracellular domain (ERBB4-ICD) [56,61]. Pharmacological inhibition of γ-secretase prevented the nuclear translocation of ERBB4-ICD [161]. Conversely, stimulating this specific intramembrane proteolysis enhanced the expression of neuroprotective genes regulated by ERBB4-ICD nuclear trafficking [56,61,161,162]. While stimulating this cleavage enhanced neuroprotective gene expression in the nucleus, the lack of specificity in γ-secretase substrates should be investigated further [56,61,161,162]. Additionally, the interaction of ErbB4-ICD with several nuclear proteins should be needed, since it provided a more precise future therapeutic approach [56,61,161,162].

Since ERBB4 mutations in ALS and FTD-ALS were associated with loss-of-function effects, gene replacement or correction became a promising therapeutic strategy [163]. In SOD1-G93A ALS mice, intracellular ERBB4 signaling fragments were depleted, signaling systemic pathway failure; however, viral overexpression of its ligand, NRG1, successfully restored pathway equilibrium and increased neuroprotective ecto-ERBB4 fragment shedding [153]. NRG1/ErbB4 signaling could regulate GABAergic transmission, and its deficiency contributed to olfactory dysfunction in AD models. Stimulating ERBB4/NRG1 signaling in the GABAergic transmission pathway improved olfactory dysfunction [75].

Zebrafish models were used to analyze ERBB4 dysfunction. Targeting ERBB4 with antisense oligonucleotides or pan-ErbB tyrosine kinase inhibitors (TKIs) resulted in different structural defects in muscle fiber development and reduced the length of motor neurons. Muscle cell-related gene expression was altered; for example, expressions of smyhc1, tpm1, or cmcl2 were reduced. However, the acetylcholine receptor expression remained normal. With muscle-specific ERBB4 expression, the mobility of zebrafish was improved. ErbB-targeting TKIs were successfully used in diverse cancers; however, their effects were opposite in ALS and motor diseases [163]. In the future, CRISPR-Cas9 will provide a precise genomic editing tool to correct ERBB4 dysfunctions. However, successful clinical application would require overcoming delivery challenges to the spinal cord and minimizing off-target effects in this multifunctional gene [163,164,165,166].

**Table 2:** Therapeutic candidates targeting ERBB4.

Therapeutic Candidate	Type	Effect	Animal Studies/Patient Studies	Reference
E4A	Small molecule, ERBB4 receptor agonist	Anti-inflammatory effects, improves mitochondria	Improved cognition, reduced amyloid deposition in mice	[101,159]
EF-1 homodimer	Small molecule, ERBB4-receptor agonist	ERBB4 stimulation	Cardioprotective effects, provided neuroprotection	[160]
neratinib/dacomitinib	Pan-ERBB inhibitors	Modulation of microglial/glial neuroinflammation	Possible prevention of secondary neurodegeneration in ALS	[145]
Masitinib	Multikinase inhibitor	Modulation of microglial/glial neuroinflammation	Slower functional decline and increased survival in ALS patients	[161]
AG1478/ siRNA	ERBB4 inhibitor	Inhibition of JNK phosphorylation	Mice: prevented amyloid pathology or Tau phosphorylation	[100]
NRG1 stimulation	Stimulating ERBB4	Increased ecto-ERBB	Mice: increased the ecto-ErbB4 fragment, neuroprotection	[153]
γ-secretase stimulator	Stimulating ERBB4-ICD	Possible effect on neuroprotection	Further studies are needed	[56,161,167]
Future CRISPR Cas9	Gene therapy	Possible replacement of the mutant ERBB4	Further studies are needed	[163,164,165,166]

### 5.2. Translational Challenges and Barriers to Clinical Implementation

A major hurdle in small-molecule drug development targeting ERBB4 could be achieving true selectivity over structural paralogs, particularly ERBB1 (EGFR), ERBB2, and ERBB3. Because active-site kinase domains across the ERBB family share high sequence conservation, small-molecule inhibitors (e.g., AG1478, neratinib) frequently inhibited EGFR and ERBB2 simultaneously. While microglial EGFR inhibition dampened reactive astrogliosis, systemic pan-ERBB inhibition disrupted epithelial homeostasis, leading to gastrointestinal toxicity and skin rash [161,167,168]. Conversely, broad agonist stimulation increased the risk of off-target cross-activation of EGFR/ERBB2 in oncogenesis [168]. Because ERBB4 and its paralogs were overexpressed in human cancers, chronic administration of central ERBB4 agonists (e.g., E4A, EF-1, recombinant NRG1) carried the potential risk of promoting peripheral cell proliferation and tumorigenesis [153,159,160,169]. Furthermore, given that NRG1–ERBB4/ERBB2 signaling should be essential for adult cardiomyocyte stress responses, non-selective pan-ERBB inhibition increased the risk of cardiotoxicity and heart failure [19,145,160,164]. Attempts to modulate ERBB4 signaling through proteolytic processing, including controlling γ-secretase-mediated cleavage to regulate 4ICD nuclear translocation, were also limited by substrate non-specificity [56,162]. Because γ-secretase cleaved several essential substrates (including Notch and APP), global inhibition or stimulation induced severe off-target effects and gastrointestinal toxicity, rendering non-selective proteolysis modulators clinically non-viable [56,162].

The functional outcome of ERBB4 signaling in neurodegeneration was not uniformly beneficial or damaging; rather, it could depend on the cell type, disease stage, and receptor processing dynamics. In the case of PV+ and motor neurons, activating ERBB4 signaling was protective by maintaining homeostasis (excitation/inhibition (E/I) balance or synaptic stability) [117,118,119,120,121,122,123,124]. In midbrain dopaminergic neurons, ERBB4 stimulation may also be protective through ferroptosis suppression under toxic stress [128,129,130,131]. However, ERBB4 activation was damaging in the case of hippocampal neurons specifically under amyloid-beta stress, where pathological cleavage generated 4ICD fragments and promoted mitochondrial mislocalization, resulting in neuronal loss through Bax/BAK-dependent apoptosis and JNK-mediated Tau phosphorylation [65,101,102,103,104,105,106]. Also, higher ERBB4 expression was reported as an early signal of AD pathology, and ERBB4 overexpression may have been impacted or affected by AD-related neurodegeneration [55]. In glial cells, the outcome depended on paralog selectivity. The distinction between ERBB1 signaling and anti-inflammatory ERBB4 activation was reflected in contrasting findings with pan-ERBB inhibitors (which blocked EGFR-driven astrogliosis) and ERBB4-selective agonists, including E4A (which suppressed microglial inflammasomes) [100,159,160,161].

While CRISPR-Cas9 genome editing and AAV-mediated *NRG1* gene delivery offered promising strategies to correct loss-of-function *ERBB4* mutations, clinical translation was constrained by delivery limitations. Achieving uniform transgene expression across human spinal cord anterior horn motor neurons required high-dose viral vector infusion, which could raise risks of systemic immunogenicity, neurotoxicity, or off-target genomic cleavage [164,165,166,167,168,169,170].

## 6. Conclusions and Limitations

Emerging evidence suggested that ERBB4-NRG1 signaling and the HPA axis were functionally interconnected regulators of stress, neuroinflammation, and neuronal survival. Together, they could maintain synaptic balance and protection against neurodegeneration. Dysregulation of either pathway resulted in increased vulnerability to neurodegeneration by impairing GABAergic and dopaminergic signaling, increasing chronic glucocorticoid exposure, promoting mitochondrial dysfunction, or enhancing inflammatory activation [25,90,171].

ERBB4 could play an important role in homeostasis and connect neuroendocrine stress regulation, local circuit stability, and central anti-inflammatory tone [157]. At the molecular level, canonical NRG1–ERBB4 activation induced the neuroprotective PI3K/Akt and MAPK cascades, while its regulated intramembrane proteolysis produced the 4ICD fragment to modulate nuclear transcription and synaptic scaffolding. At the circuit level, ERBB4 enrichment in PV+ fast-spiking interneurons was indispensable for maintaining cortical and hippocampal excitation/inhibition (E/I) balance and maintaining GABAergic inhibition over hypothalamic corticotropin-releasing hormone (CRH) release [117,118,119,120,121,122,123,124,172,173]. Disruption of this system, whether through genetic loss-of-function variants (e.g., in ALS19/FTD-ALS), aberrant overexpression of ERBB4, impaired neurodevelopmental wiring, or chronic hypercortisolemia and amyloid/tau pathology (e.g., AD), compromised neural resilience, accelerated neuroinflammation, and created a feed-forward neuroendocrine/neurodegenerative failure loop [55,102,103].

Emerging evidence suggested that ERBB4-NRG1 signaling operated at the intersection of local circuit physiology, neuroinflammation, and systemic stress responses. However, a central unresolved question was whether ERBB4 dysfunction acted as a primary driver of disease etiology or as a secondary, compensatory response to chronic stress and neuroinflammatory signaling [121,158,174]. Although anatomical mapping demonstrated ERBB4 expression across key HPA axis regulatory hubs, the direct functional evidence linking ERBB4 dysfunction to primary HPA axis failure in neurodegeneration remained limited. Studies in Nrg1-deficient and Esr2 knockout rodent models confirmed [86,87,88,89,90,91,92] that impaired ERBB4 signaling correlated with aberrant ACTH and corticosterone dynamics. This interaction was hypothesized to be associated with the loss of ERBB4-dependent GABAergic inhibition onto CRH-releasing neurons and an impaired receptor network [85,86,87,88]. However, these observations were made based on constitutive knockout or acute stress models. Additional studies were needed to determine whether hypothalamic ERBB4 disruption initiated HPA breakdown or acted as a secondary downstream marker of broader neuroinflammatory degeneration in human AD, ALS, or PD. Thus, the proposed role of ERBB4 as a causal bridge between HPA dysregulation and neurodegenerative pathology may be a promising but largely speculative working hypothesis that requires additional targeted conditional cell-type knockout studies [85,86,87,88,89,90,91,92,93].

A central unresolved question could be whether hypothalamic ERBB4 dysfunction acted as a primary initiator of HPA axis breakdown or it could be a secondary consequence of chronic neuroinflammation and age-dependent endocrine decline [25,90,121,174,175]. While Esr2-/-mice demonstrated a clear link between ER-beta, ERBB4 depletion, and HPA axis hyperresponsiveness, researchers should be careful when extrapolating these complete germline knockouts to human age-related neurodegeneration. In humans, age-related decline in estrogens occurred gradually alongside cell-type-specific receptor shifts [172,173]. Thus, whether hypothalamic ERBB4 downregulation became a primary driver of HPA axis dysregulation or a secondary consequence of broader age-dependent endocrine failure remained an open question in human post-mortem validation studies [83,84,85,86,87,88,176].

Additional limitations included significant variation in the reproducibility and cohort robustness of key ERBB4 findings across neurodegenerative diseases. Circuit-level phenomena, including ERBB4-mediated regulation of GABAergic fast-spiking interneurons, synaptic plasticity, and dopaminergic survival, showed high reproducibility across diverse knockout rodent lines and independent laboratories [78,79,80]. However, the human genetic and biomarker findings require further replication, since most FTD-ALS variants were reported in single cases or one family. Both genetic studies and plasma studies required standardized multi-center genetic registries, and larger CSF/plasma validation cohorts will be essential to establish the true reproducibility and diagnostic utility of ERBB4 alterations in human disease [126,153,158]. A critical unresolved question was whether ERBB4 dysfunction would be a driver of disease or a compensatory response to chronic stress and neuroinflammatory signaling. The apparent paradox, where both ERBB4 activation and inhibition showed context-dependent neuroprotective effects, highlights the necessity of cell type-specific and disease stage-specific therapeutic strategies [18,172].

Several promising therapeutic candidates were identified, which targeted the ERBB4-related pathways. Small-molecule agonists, such as 4-bromo-1-hydroxy-2-naphthoic acid (E4A), were suggested as promising therapeutic candidates. By selectively activating ERBB4 without cross-activating paralogs, E4A suppressed microglial TLR4/NFkappaB/NLRP3 inflammasome activation, restored mitochondrial DOCK3/SIRT3 signaling, and preserved synaptic integrity in AD models [47,101,159]. Targeted restoration of ERBB4-dependent GABAergic tone or interneuron progenitor transplantation also provided a circuit-level approach to correct local E/I imbalances and suppress hyperactive HPA feedback loops [75,163]. Impaired ERBB4-ICD impacted different neurodegenerative diseases, including AD, PD, ALS and schizophrenia, through diverse mechanisms, even though targeting ERBB4-ICD became a promising approach for neuroprotection [60,61,62].

Translating preclinical findings from animal models of ERBB4 dysregulation to human clinical pathology was also a significant challenge due to fundamental physiological and methodological divergences. While transgenic and knockout rodent models (e.g., APP/PS1, conditional ErbB4 KO) were pivotal in elucidating cell-autonomous and circuit-level mechanisms, such as parvalbumin-positive interneuron degeneration and E/I imbalance, they imperfectly recapitulate human disease etiology [15,145]. In mouse or rat models, experiments were performed using either protein overexpression or complete biallelic gene knockouts. In contrast, patients with ALS19 and FTD-ALS carried heterozygous ERBB4 mutations, and the disease progressed over decades in an aging nervous system [15,177]. In ALS/FTD, ERBB4 mutations may have loss-of-function mechanisms; the brain may lose the essential survival/protective signaling pathway in neurons [15,177]. However, in AD, ERBB4 pathology was associated with pathological mislocalization; ectopic expression of ERBB4 in excitatory neurons may result in hyperactivation and toxic immune/synaptic cascades [55]. These contrasting profiles indicated a cell-type-dependent dual role for ERBB4 in neurodegeneration, which became a major challenge for ERBB4-targeted therapies [55].

Furthermore, acute neurotoxin models of PD (e.g., MPTP or 6-OHDA) demonstrated strong ERBB4-mediated neuroprotection against sudden mitochondrial insult, but did not properly reflect the chronic, progressive α-synucleinopathy of PD [127,128,129,130]. Additionally, neuroendocrine interactions were different in rodents and humans; rodent stress responses were stimulated by corticosterone and did not clearly reflect the human HPA axis aging dynamics and cortisol kinetics [178]. Validation of animal-model findings in human patient-derived iPSC cells, cerebral organoids, and longitudinal post-mortem cohorts is essential to establish true clinical relevance [178].

As discussed above, ERBB4 therapy for neurodegenerative diseases is also challenging. For example, while exogenous NRG1 administration or small-molecule ERBB4 agonists (e.g., E4A) resulted in neuroprotective and anti-inflammatory effects in AD models, post-mortem human AD tissue showed elevated ERBB4 expression and co-localization with pro-apoptotic Bax, while reported Aβ–ERBB4 interactions stimulated JNK-mediated Tau phosphorylation [101,109]. This paradox was associated with receptor cleavage state and cellular compartmentalization. Under physiological conditions, full-length membrane-bound ERBB4 induced the PI3K/Akt and anti-inflammatory cascades and supported synaptic stability. However, under chronic amyloid or stress-induced processing, cleavage by TACE and γ-secretase generated the soluble 4ICD fragment. While nuclear 4ICD translocation regulated gene transcription, excessive 4ICD mistargeting to the outer mitochondrial membrane directly repressed Bcl-2 and promoted BAK oligomerization, converting a neuroprotective receptor into a pro-apoptotic executioner [58,59,60,101]. Similarly, the conflict between therapeutic ERBB4 activation in neurons and therapeutic ERBB inhibition (by TKIs such as neratinib or masitinib) reflected cell-type-specific receptor distribution. While the neuronal ERBB4 activation maintained E/I balance and survival, the pan-ERBB inhibitors targeted the reactive glia to dampen hyperactive EGFR (ERBB1) signaling, which was reported to exacerbate neuroinflammatory neurodegeneration [55,159,160,161,162,163,164,165,166]. Dissecting these context-, stage-, and cell-type-specific outcomes is essential to prevent conflicting preclinical reports from stalling translational development.

Taken together, ERBB4 is a relatively new target, which may represent a molecular bridge linking stress endocrinology and neurodegenerative vulnerability across the spectrum of neurodegenerative diseases. Understanding these intersections may open new avenues for biomarker development and targeted neuroprotective therapies. However, given the multifunctional and context-dependent nature of ERBB4 signaling, therapeutic interventions require careful stratification by disease type, mutation status, and neuroendocrine profile. Further studies are needed to investigate the ERBB4-HPA axis, hormone and stress regulation, biomarker (including ERBB4 in plasma and CSF), and multi-omics studies [25,90,121,174,175].

## Figures and Tables

**Table 1:** Mutations in ERBB4, discovered in patients diagnosed with FTD-ALS or pure ALS.

Mutation	AOO	Diagnosis	Symptoms	Family History	Other Remarks	References
Tyr86His	52	FTD-ALS	Behavioral impairment, motor impairment	NA	Co-existed with GRN Pro451Leu, possible common pathways	[124]
Arg106His	57	ALS	Spinal ALS	Negative	NA	[118]
Gln164Pro	43	ALS	Spinal ALS	Negative	NA	
Val212Leu	48	ALS	Bulbar ALS	Negative	NA	
Ile658Phe	38	ALS	Dysphonia, dysarthria, and asthenia, normal cognition	Negative	NA	[125]
Ile666Thr	NA	FTD-ALS	Bulbar ALS	NA	NA	[118]
Asn706Asp	53	ALS	Motor impairment	Negative	Reduced the phosphorylation of ERBB4	[119]
Ile712Met	55	FTD-ALS	Personality and memory changes, motor impairment	Incomplete penetrance	Reduced phosphorylation of ERBB4	[121]
Arg782Pro	43	ALS	Limb involvement	Negative	NA	[126]
Met799Thr	38	ALS	Limb involvement	Negative	NA	[126]
Arg927Gln	60s	Late onset ALS	Motor impairment, possible locked-in in late disease stages	Positive	ERBB4-NRG1 pathway disruption	[117,122]
Ser997Arg	51	ALS	Limb involvement	Negative	NA	[126]
Arg1112His	NA	FTD-ALS	NA	NA	NA	[111]
Arg1275Trp	45	ALS	Upper limb involvement	NA	No cognitive impairment	[117]
c.1490-3C > T	60s	68	Limb involvement	Negative	Reduced ERBB4 expression	[126]

## Data Availability

No new data were created or analyzed in this study.
